# Special Issue: Agricultural Nanotechnology

**DOI:** 10.3390/plants13040489

**Published:** 2024-02-08

**Authors:** Kamel A. Abd-Elsalam

**Affiliations:** Plant Pathology Research Institute, Agricultural Research Centre, Giza 12619, Egypt; kamelabdelsalam@gmail.com

**Keywords:** green synthesis of nanoparticles, nanofertilizer, nanopesticide, food processing and packaging, post-harvest treatment, nanotoxicity, regulations, biosafety

## Abstract

Agricultural nanotechnology has considerable promise for addressing global agricultural production/security, biodiversity, and global warming issues. Current trends in publications and patents demonstrate that biotechnology technologies, particularly for crops, are being developed to improve agricultural productivity and disease management. In the current issue, we strongly advocate for the use of biosynthesized nanoparticles from a variety of sources, including plants, agricultural waste, and microbes, as a prerequisite for significant and in-depth study. Nanomaterials offer a wide range of practical uses in agriculture, including nanofertilizers, nanopesticides, nanoherbicides, nanosensors, and smart delivery systems for controlled agrochemical release. Additionally, nano-tools are employed for plant breeding and genetic manipulation. A thorough examination of the physicochemical soil properties of the agricultural fields where nanoparticles will be used will aid in minimizing their impact on plant and soil biota. Finally, and most importantly, we strongly recommend the inclusion of nanotoxicity, legislation, biosafety, and risk assessment as the top priorities when developing regulatory policies to address biosafety concerns. Starting today, thorough efforts must be carried out to advance and develop futuristic work based on recognized knowledge shortages.

## 1. Introduction

Agri-nanotechnology, which combines nanoscience and agriculture, has emerged as a transformative field with far-reaching implications for sustainable farming techniques and global food security [1]. Agricultural nanotechnology is the use of nanomaterials and nanotechnology in many parts of agriculture to improve crop yield, soil quality, and sustainable farming practices. This technology has a wide range of applications, including nanosensors for monitoring plant health and nanofertilizers for effective nutrient delivery [2]. Precision farming is a critical application in which nanoscale sensors and monitoring devices give real-time data on soil conditions, crop health, and environmental parameters. This allows farmers to maximize resource consumption, save water and fertilizer, and reduce environmental impact [3]. Crop protection is significantly influenced by nanomaterials, including nanoparticles and nanocomposites [4]. Nano-enabled agrochemicals offer improved effectiveness, precise delivery, and regulated release, resulting in more efficient management of pests and diseases. By adopting this approach, the environmental hazards associated with conventional agrochemicals can be reduced, while promoting healthier crop growth [5].

Furthermore, nanoscale delivery technologies contribute to biofortification, which addresses nutritional issues. Nanoencapsulation allows for the controlled release of vital nutrients, ensuring optimal absorption by plants and, as a result, better nutritional value in crops [6]. In precision agriculture, nanoscale imaging tools and sensors allow for comprehensive monitoring of plant health and environmental conditions. This data-driven strategy enables farmers to make more educated decisions, promotes sustainable farming methods, and reduces agriculture’s environmental imprint [7]. Agricultural nanotechnology also transforms seed treatments and genetic alterations. Nanoparticle coatings boost seed germination, promote root development, and provide early disease defense. Furthermore, nanogenomics enables precise genetic alterations, which optimize plant properties for greater resilience, productivity, and nutritional value [8].

Agricultural nanotechnology challenges and considerations include potential environmental impact, regulatory frameworks, and public acceptance. While technology has the potential to revolutionize agriculture, careful consideration of its long-term implications is required to ensure sustainable and safe deployment [9]. However, agricultural nanotechnology, like any other developing technology, raises worries about potential environmental and safety risks. Robust regulatory frameworks and ethical concerns are essential for guaranteeing responsible development and implementation [10]. To summarize, agricultural nanotechnology represents a paradigm change in modern farming, providing creative answers to critical issues. By leveraging nanoscience, this field presents the potential of sustainable, efficient, and resilient agriculture practices to satisfy the demands of a growing global population.

## 2. Published Articles in the Current Issue

Zinc oxide nanoparticles (ZnONPs) were produced utilizing an environmentally benign and sustainable method by extracting discarded acid lime (*Citrus aurantifolia* Swingle) peels. These nanoparticles were subsequently used to create an edible nanocoating for acid lime fruits by combining them with a carboxymethyl cellulose (CMC) matrix. The nanocoating films’ morphological, physicochemical, and antibacterial properties were all carefully investigated and assessed. This study also investigated how the postharvest dipping of fruits in different edible coatings and preharvest spraying with potassium from organic and mineral sources affect the quality and shelf life of acid lime fruits. Comparing the preharvest spraying of potassium tartrate and potassium thiosulfate to the control treatment, the results show that potassium tartrate considerably improved fruit characteristics (Contribution 1).

Silver nanoparticles (Ag-NPs) were produced in an ecofriendly manner through the process of green synthesis, utilizing an extract derived from *Ocimum basilicum* plant. The Ag-NPs underwent characterization using various techniques, including Scanning Electron Microscopy (SEM), Dynamic light scattering (DLS), Transmission electron microscopy (TEM), Energy Dispersive X-ray (EDX), Fourier-transform infrared spectroscopy (FTIR) and zeta potential distribution methods. The study additionally investigated the antiviral activity and efficacy of the biosynthesized Ag-NPs in inducing systemic acquired resistance (SAR) against CMV. Furthermore, the impact of Ag-NPs on plant growth parameters, CMV accumulation levels, antioxidant enzymes, and the transcriptional levels of defense-related genes was examined. Notably, greenhouse experiments demonstrated that the application of Ag-NPs (100 µg/L) as a foliar treatment on squash plants induced SAR, resulting in reduced disease severity and up to a 92% decrease in CMV accumulation levels. The utilization of Ag-NPs as a potential inducer for systemic resistance in squash against CMV infections presents a promising alternative technique for managing plant viral diseases without the need for pesticides (Contribution 2).

The purpose of this work was to examine the preventive effects of iron (Fe) and zinc (Zn) nanoparticles (NPs) in reducing stress symptoms in Basella alba seedlings produced by lead (Pb) exposure. The results imply that seed priming with Zn or Fe NPs may be a more successful strategy for reducing Pb stress, particularly in the early phases of seedling growth. Future studies will concentrate on examining how both types of NPs work in concert to promote seed germination and seedling growth (Contribution 3).

This study aimed to investigate the beneficial effects of applying selenium nanoparticles (Se-NPs) to the leaves on specific morphological and phytochemical characteristics of the plant under varying levels of salinity stress (0, 30, 60, and 90 mM NaCl), given the significant medicinal importance and economic value of pineapple mint. The findings showed that there were no appreciable variations in the dry weight of pineapple mint caused by either salt stress or foliar treatment of Se-NPs. Nonetheless, the application of Se-NPs at the proper concentration led to a rise in certain of the constituents of the pineapple mint essential oil (Contribution 4).

The aim of this study was to assess the impact of chitosan/dextran nanoparticles (CDNPs) on the induction of systemic acquired resistance (SAR) and the transcriptional levels of defense-related genes, including peroxidase (POD), pathogen-related protein-1 (PR-1), and phenylalanine ammonia-lyase (PAL). Additionally, the study examined the effects of CDNPs on total carbohydrate and total phenolic content. Under greenhouse conditions, the foliar application of CDNPs at a concentration of 100 µg mL^−1^ resulted in a reduction in the severity of viral diseases, the induction of SAR, decreased accumulation levels of AMV (the virus examined), and an up-regulation of the transcriptional levels of POD, PR-1, and PAL genes (Contribution 5). 

Gold nanoparticles (PtubAuNPs) were synthesized using *Polianthes tuberosa* flower filtered extract as both a reducing and stabilizing agent. The antibacterial activity of PtubAuNPs was assessed using the agar well diffusion method, and the results showed significant antagonistic activity against the tested pathogens. Additionally, the cytotoxicity of PtubAuNPs was evaluated in MCF 7 cells through the MTT (3-(4,5-dimethylthiazol-2-yl)-2,5-diphenyltetrazolium bromide) assay. The study demonstrated that PtubAuNPs induced toxicity in MCF 7 cells in a dose-dependent manner, with a minimum concentration of 100 µg/mL, by promoting apoptosis. In conclusion, the findings highlight the potential of PtubAuNPs as a powerful nanomaterial that can serve as an effective antimicrobial and anticancer agent (Contribution 6). 

Morcia-Morales et al. conducted a study to examine the impact of multi-walled carbon nanotubes (MWCNTs) on the in vitro multiplication of sugarcane (*Saccharum* spp.) using a temporary immersion system. The researchers performed a morphological characterization of MWCNTs using a transmission electron microscope. Different concentrations of MWCNTs (0, 50, 100, 200 mg L^−1^) were added to the Murashige and Skoog liquid culture medium during the multiplication stage. The results indicated that low concentrations of MWCNTs had positive physiological effects on the development of sugarcane in the in vitro multiplication stage when using temporary immersion bioreactors. This suggests that MWCNTs can induce a hormetic effect during the in vitro shoot multiplication of sugarcane and may have potential applications in other plant species. Additionally, MWCNTs offer opportunities to enhance crop production by improving efficiency during micropropagation (Contribution 7).

Abdelhameed and his team investigated the effects of ZnFe_2_O_4_ NPs on Arbuscular Mycorrhiza (AM) fungal colonization and their combined influence (AM fungi and ZnFe_2_O_4_ NPs) on the growth performance and chlorophyll content of green pea plants. The study also examined Zn and Fe concentrations and translocation in plants treated with AM and non-AM fungi. Structural and magnetic properties of ZnFe_2_O_4_ NPs were initially characterized due to the impact of NPs being dependent on their concentration, size, and distribution. The results demonstrated that ZnFe_2_O_4_ NPs had a positive effect on pea plants compared to the control, with further improvements observed in plants inoculated with AM fungi. Additionally, ZnFe_2_O_4_ NPs were taken up by the roots and translocated to the leaves, resulting in enhanced mineral uptake and various plant growth parameters without causing phytotoxic effects (Contribution 8).

The magnetic separability and reusability of ferrites and their GO composites were investigated for the cyclic degradation of pollutants. GO-doped metal ferrites (GO-Fe_3_O_4_ and GO-CoFe_2_O_4_) were prepared and characterized using scanning electron microscopy, X-ray diffraction, and Fourier-transform infrared spectroscopy. The photocatalytic potential of the catalysts was assessed for the degradation of acetamiprid (Contribution 9).

Metal oxide nanoparticles offer several advantages as fungicides, including increased efficacy, reduced environmental impact, and lower application frequencies. Hybrid nanoparticles that combine different metal oxides, such as copper oxide and zinc oxide, have the potential to exhibit synergistic effects for enhanced antifungal activity. This review article discusses the role of mono-, bi-, and tri-metal oxide nanoparticles in controlling phytopathogenic fungi in sustainable agriculture. The challenges and future directions of applying metal oxide nanoparticles as potential antifungal agents in sustainable agriculture are also discussed (Contribution 10).

## 3. Conclusions

Agricultural nanotechnology uses nanomaterials and nanotechnology to enhance crop production, improve soil quality, and mitigate environmental impacts. It enables the development of nanofertilizers, nanopesticides, nanosensors, and nanoremediation techniques to improve nutrient uptake, pest management, and plant health monitoring. Nanotechnology also aids in precision agriculture through nanoscale sensors and imaging technologies. However, challenges include safety concerns, a lack of standardized regulations, and public perception concerns regarding the use of nanotechnology in agriculture.
